# CDH1 methylation in preoperative peritoneal washes is an independent prognostic factor for gastric cancer

**DOI:** 10.1002/jso.23116

**Published:** 2012-04-18

**Authors:** Qi-Ming Yu, Xin-Bao Wang, Jun Luo, Shi Wang, Xian-Hua Fang, Jiang-Liu Yu, Zhi-Qiang Ling

**Affiliations:** 1 Zhejiang Cancer Research Institute, Zhejiang Province Cancer HospitalZhejiang Cancer Center, Banshanqiao District, Hangzhou, China; 2 Department of Tumor Surgery, Zhejiang Province Cancer HospitalZhejiang Cancer Center, Banshanqiao District, Hangzhou, China; 3 Department of Endoscopy, Zhejiang Province Cancer HospitalZhejiang Cancer Center, Banshanqiao District, Hangzhou, China; 4 Department of Pathology, Zhejiang Province Cancer HospitalZhejiang Cancer Center, Banshanqiao District, Hangzhou, China

**Keywords:** CDH1, gastric cancer, methylation, preoperative peritoneal washes (PPW), prognosis, tumor progression

## Abstract

**Background and Objectives:**

To investigate the clinical value of CDH1 methylation in preoperative peritoneal washes (PPW) from gastric cancer patients.

**Methods:**

CDH1 methylation was detected by real-time methylation specific-PCR in tumor tissues and corresponding PPW from 92 gastric cancer patients, gastric mucosa from 40 chronic gastritis patients and 48 normal persons.

**Results:**

CDH1 methylation was found in 75 of 92 (81.5%) gastric cancer tissues, which significantly correlated with size, growth pattern, differentiation, lymphatic invasion, venous invasion, invasion depth, lymph node metastasis, distant metastasis, and TNM stage of tumor (all *P* < 0.05), but its relationship to age, gender, tumor site, and *H. pylori* infection was not found (all *P* > 0.05). The percentage of CDH1 methylation in PPW was 48.9%, of which the Aζ value of ROC curve was 0.8 compared to that in gastric cancer tissues. Kaplan–Meier analysis showed that there was a significant difference in disease-free survival (DFS) between the patients with or without methylated CDH1 in their PPW (χ^2^ = 109.64, *P* < 0.000). Cox regression analysis revealed CDH1 methylation in PPW was an independent risk factor for gastric cancer patients, with a remarkable decrease in DFS after postoperative 30 months.

**Conclusions:**

Methylated CDH1 in PPW predicts poor prognosis for gastric cancer patients. J. Surg. Oncol. 2012; 106:765–771. © 2012 Wiley Periodicals, Inc.

## INTRODUCTION

Gastric carcinoma (GC) is one of the highly prevalent malignant diseases worldwide which carries a very poor prognosis, and the 5-year survival rate is low. GC is also one of the most common digestive tract cancers in China, with a high incidence and mortality, approximately accounting for 10% of malignancies 1. It has been already verified that GC was characterized by rapid deterioration, early metastasis, without much chance for radical operation and poor prognosis. Those patients without non-specific symptoms at early stage had mostly lost the opportunity of surgical therapy when gastric cancer was detected at advanced stage 2, 3. So, new molecular biomarkers are undoubtedly needed to facilitate the early diagnosis, metastasis, and prognosis evaluation for GC.

Accumulating evidence indicates that aberrant promoter methylation is one of the most common molecular alterations in human neoplasia, and considered as a sensitive and very promising biomarkers in early diagnosing of tumors 4, 5. Furthermore, tumor cells can release DNA to peripheral blood and enriched circulating DNA level can be found in the serum of cancer patients, several times higher than normal. Previous studies showed that methylation of multiple genes were detected in blood plasma, urine, sputum and peritoneal washes in several different cancers, and high frequent hypermethylation of suppressor was mostly cancer specific, so it may be utilized as a molecular diagnostic marker of cancer 6–11.

CDH1, a tumor metastasis suppressor gene, is located on chromosome 16q22.1. Its product is a Ca^2+^-dependent cell adhesion molecule, and mutations in this gene have been associated with the origin, development, invasion, metastasis, and prognosis of carcinomas derived from a variety of epithelial tissues 12, 13. The product of CDH1 expression, E-cadherin, mediates the adhesion reaction between the same types of cells and plays a role in the cytoskeleton, implying that the degree of its expression and function directly impact the detachment and re-attachment of tumor cells. When the activity of CDH1 is normal, tumor cells are not easily detached from the primary tumor. On the contrary, CDH1 inactivation results in decreased cell adhesion and abnormal polarity, which promotes tumor metastasis 13–16. CDH1 methylation-modulated loss of gene expression has been shown to be important in the origin and development of many tumors 17–23. Various degrees of methylation in the CDH1 promoter CpG islands and the consequent loss of E-cadherin expression were reported in many tumor tissues, such as cervical carcinoma 17, prostate carcinoma 18, malignant melanoma 19, non-small cell lung carcinoma 20, liver carcinoma 21, esophageal cancer 22, and GC 23. E-cadherin expression was also shown to be associated with the level of methylation of promoter CpG islands, implying that this mechanism may be an early event in the malignant process, and that it is an important event in tumor occurrence and development 17–24. Here, we used real-time methylation specific-PCR (real-time MSP) assays to detect the methylation status of CDH1 promoter 5′-CpG island in preoperative peritoneal wash DNA from 92 patients with GC, in order to evaluated its prognostic potential for GC patients.

## MATERIALS AND METHODS

### Patients and Tissue Samples

The Institutional Review Board on Medical Ethics, Zhejiang Province Cancer Hospital approved the method of tissue collection including informed consent from all patients. The present study analyzed the gastric cancer tissue and the corresponding preoperative peritoneal wash of 92 cases. Tumor tissues were collected at the time of surgery from 92 patients with primary GC at Zhejiang Province Cancer Hospital, Zhejiang Province People's Hospital and the First People's Hospital of Chunan County from January 2008 to December 2009. The diagnosis of all patients without preoperative radiotherapy or chemotherapy was confirmed not only by gastrointestinal endoscopy followed with pathological analysis but also by examination of the paraffin embedded tissue sections. Demographic, clinical and histopathological parameters are shown in Table I. As a measure of prognosis, we analyzed the clinical data concerning disease-free survival (DFS), defined as the time from surgery data to first recurrence or death by gastric cancer or last contact. The followed-up was carried out by our study group members and ended on June 30, 2011. Meanwhile, 40 specimens of normal gastric mucosal without GC or other digestive system diseases individuals (male: n = 24, female: n = 16; mean age: 51 years) and 48 specimens from patients with chronic gastritis were also collected at Zhejiang Province People's Hospital, Fifth People's Hospital at Hangzhou Yuhang District and the Hospital of Traditional Chinese Medicine (TCM) at Hangzhou Yuhang District. In the normal control group, three biopsies were captured by endoscopy from each individual's gastric antrum, the first biopsy for rapid urease test, the second were immediately frozen and stored, the third for paraffin embedded section.

**TABLE I:** Clinicopathological Correlations of CDH1 Hypermethylation in Gastric Cancer Tissues

		CDH1			
Clinicopathological parameters	n	M	U	χ^2^	*P*-value
Gender				1.875	0.171
Male	54	47	7		
Female	38	38	10		
Age				0.120	0.730
<60	68	56	12		
≥60	24	19	5		
*H. pylori*				4.113	0.043
(−)	50	37	13		
(+)	42	38	4		
Localization				0.097	0.755
Cardia	30	25	5		
Body/antrum	62	50	12		
Tumor size (cm)				7.678	0.006
<5	60	44	16		
≥5	32	31	1		
Growth manner				0.039	0.843
Swell type	20	16	4		
Infiltration type	72	59	13		
Histological grade				6.778	0.009
High/medium	62	46	16		
Low	30	29	1		
Lymphatic invasion				7.781	0.005
(−)	67	50	17		
(+)	25	25	0		
Venous invasion				4.065	0.044
(−)	69	53	16		
(+)	23	22	1		
Invasive depth				47.164	0.000
T1	17	4	13		
T2	21	19	2		
T3	35	33	2		
T4	19	19	0		
Lymph node metastasis				12.195	0.000
N0	46	31	15		
N1–3	46	44	2		
Distant metastasis				0.646	0.421
M0	85	68	17		
M1	7	7	0		
TNM stage				48.803	0.000
I	17	4	13		
II	29	25	4		
III	37	37	0		
IV	9	9	0		

### Preoperative Peritoneal Washes (PPW)

About 200 ml of warm normal saline were introduced and manually dispersed in the Douglas cavity, para-colic gutters and in the right and left subphrenic cavity, when entering the abdominal cavity, prior to manipulating the tumor. At least 100 ml of fluid was subsequently recovered, after gentle stirring, from several regions of the abdominal cavity. The fluid was then centrifuged for 10 min at 1,500 rpm. The sediment was smeared onto one or more glass slides and stained using the Papanicolau's method. All cytological examinations were performed by three cytopathologists independently. Cytological findings were classified as positive or negative. The following cell characteristics were used to determine the presence of malignant cells: presence of aggregate, size, shape, type of cytoplasm, cytoplasmic vacuoli, mainly nuclear abnormalities, nuclear chromatin, nuclear cytoplasmic ratio, mitotic figures, and nucleolar prominence. Meanwhile, we isolated cells using some preoperative peritoneal washes of all patients, and immediately stored these cells samples at −80°C until DNA was extracted.

### Analysis of *Helicobacter pylori* (*H. pylori*) Infection

Biopsies were obtained from all patients who had endoscopic evaluations. *H. pylori* status had been determined by rapid urease test and Giemsa staining method. Urease test was done by a freshly prepared solution of urease test reagent and the test was recorded as positive if the color of the solution turned pink within 24 hr. Biopsy materials embedded in paraffin were stained with Giemsa for histopathologic examination. It was considered as *H. pylori* infection when two tests were positive, and the result with single positive was been excluded.

### Genomic DNA Extraction

Serial 5-µm sections that contained carcinoma and non-neoplastic tissues were mounted on non-coated glass slides and dried at 37°C overnight. After deparaffinization and staining with hematoxylin and eosin (H&E), we collected 5,000 nuclei from 5 to 10 serial sections using a 27G needle. The collected nuclei were treated with 40 µl of 200 µg/ml proteinase K (Sigma-Aldrich Co., St. Louis, MO) at 42°C, for 72 hr. The paramagnetic bead technology (AxyPrep Mag Blood gDNA kit, Axygen Scientific, Inc., Union City, CA) was utilized to isolate genomic DNA from fresh PPW according to kit's protocol. The protocol consists of the following step: lysis, binding, washing, and elution. Contaminants are removed during the binding and washing steps. The quality of DNA was assessed by the A260/280 ratio at NanoDrop ND-1000 spectrophotometer (NanoDrop Technologies, Inc., Wilmington, DE), DNA integrity was checked by denaturing agarose gel electrophoresis.

### DNA Bisulfite Modification and Real-Time Methylation Specific-PCR Analysis

DNA was modified by sodium bisulfite and purified and recycled according to the EpiTect Bisulfite kit (Qiagen, Inc., Gaithersburg, MD) instructions. The CDH1 methylation (M) and non-methylation (U) specific primer sequences were as follows: CDH1 (M): (F) 5′-TTA GGT TAG AGG GTA TCG CGT-3′, (R) 5′-TAA TTT TAG GTT AGA GGG TTA TTG T-3′, and the amplification length was 116 bp; CDH1 (U): (F) 5′-TAA CTA AAA ATT CAC CCC TAC CCC GAC-3′, (R) 5′-CAC AAC CCC AAT CAA CAA CAC A-3′, and the amplification length was 97 bp (GeneBank No. L34545). The primers were synthesized by Invitrogen, Carlsbad, CA. Modified DNA with sodium bisulfite was analyzed by real-time MSP on the ABI 7500 PCR (Applied Biosystems, Carlsbad, CA) instrument. The procedure was performed following the instructions for the SYBR Premix Taq ExTaq kit (TaKaRa Bio, Otsu, Japan). The quantitative methylation analysis of samples was carried out using methylation and non-methylation specific primers, respectively. The percentage of methylated DNA in the samples were calculated according to the CT value and a standard curve. The methylation percentage was calculated according to a previous report with small modification 22, 25. The methylation percentage was calculated as follows: M% = 100 × (copy number of methylated DNA/the sum of the copy number of methylated and unmethylated DNA). The sum of the copy numbers of methylated and unmethylated DNAs was used as the total copy number of DNA of the target genes. Methylated DNA was scored according to M% (0: M% < 20.0; 1: 20.0 < M% < 40.0; 2: 40.0 < M% < 60.0; 3: 60.0 < M% < 80.0; 4: M% > 80.0). 0, 1–3, and 4 were considered as unmethylated (U), partially methylated (U/M), and fully methylated (M), respectively. PCR products were separated by 2.0% agarose gel electrophoresis, visualized by EB staining, then observed and photographed under UV illumination (BioSpectrumAC BioImaging Systems; Ultra-Violet Products, Inc., Upland, CA). Human genomic DNA (NEB) treated by SssI methyltransferase in vitro was used as a positive control. Peripheral blood DNA of healthy untreated subjects was used as a negative control.

### Statistical Analysis

SPSS 17.0 (SPSS, Chicago, IL) statistical software was utilized. Comparison of incidence rate between groups was carried out using χ^2^ test and those not in accord with χ^2^ test conditions were analyzed using Fisher's exact test. DFS was calculated with the Kaplan–Meier method and significant levels were assessed by means of the log-rank test. Multivariate analysis with the Cox regression model was used to estimate the prognostic effect of methylated genes and significant levels were assessed by means of Wald test. Statistical significance was accepted at *P* < 0.05.

## RESULTS

### Methylated Status of CDH1 Gene Promoter 5′-CpG Island in GC Tissues, Cancer-Adjacent Normal Tissues, Chronic Gastric and Normal Mucous Tissues

CDH1 methylation was not found in DNA samples of the gastric mucosa from 48 patients with chronic gastritis and 40 normal persons. However, aberrant CDH1 methylation was found in 75 (81.5%) of 92 GC patients. Of these 75 patients, 45 (48.9%) were fully methylated and 30 (32.6%) were partially methylated. In the samples from cancer-adjacent normal tissues, 4 (4.3%) of 92 specimens were partially methylated (U/M) in CDH1 gene promoter. These two levels were significantly different (*P* < 0.01).

### Relationship Between CDH1 Methylation in GC Tissues and Clinicopathologic Parameters

The rate of CDH1 methylation was 63.0% (29/46) in GC tissues from the patient in stage I/II, while was 100.0% (46/46) in stage I/II, with a significant difference between these two groups (χ^2^ = 20.853, *P* < 0.001). Moreover, the rate of CDH1 methylation was 95.7% (44/46) in GC tissues with lymph node metastasis, while was 67.4% (31/46) in GC tissues without lymph node metastasis, also with a significant difference between these two groups (χ^2^ = 12.195, *P* < 0.001). The correlations between CDH1 methylation in GC tissues and clinicopathologic parameters are listed in Table I.

### Detection of CDH1 Methylation in PPW From GC Patients

Of 92 GC patients, 45 (48.9%) cases were fully methylated, 30 (32.6%) cases were partially methylated and 17 (18.5%) cases were unmethylated in the 5′-CpG island of CDH1 promoter. Based on the definition that considered both fully and partially methylated as aberrant methylation, we found the rate of aberrant CDH1 methylation was 81.5% (75/92), while the rate of unmethylation was 18.5% (17/92).

### Correlation Between CDH1 Methylation in PPW From GC Patients and Clinicopathologic Parameters

It was found that CDH1 methylation correlated significantly with invasion depth, lymph node metastasis, distant metastasis and TNM stage of tumor (all *P* < 0.05), but its relationship to age, gender, tumor site and infection of *H. pylori* was not found (all *P* > 0.05). Correlations between CDH1 methylation in preoperative peritoneal washes from GC patients and clinicopathologic parameters are listed in Table II.

**TABLE II:** Clinicopathological Correlations of CDH1 Hypermethylation in Preoperative Peritoneal Washes

		CDH1			
Clinicopathological parameters	n	M	U	χ^2^	*P*-value
Gender				0.452	0.501
Male	54	28	26		
Female	38	17	21		
Age				1.693	0.193
<60	68	36	32		
≥60	24	9	15		
*H. pylori*				1.058	0.304
(−)	50	22	28		
(+)	42	23	19		
Localization				0.348	0.555
Cardia	30	16	14		
Body/antrum	62	29	33		
Tumor size (cm)				7.727	0.005
<5	60	23	37		
≥5	32	22	10		
Growth manner				19.721	0.000
Swell Type	20	1	19		
Infiltration type	72	44	28		
Histological grade				21.106	0.000
High/medium	62	20	42		
Low	30	25	5		
Lymphatic invasion				35.854	0.000
(−)	67	20	47		
(+)	25	25	0		
Venous invasion				26.810	0.000
(−)	69	23	46		
(+)	23	22	1		
Invasive depth				37.442	0.000
T1	17	0	17		
T2	21	6	15		
T3	35	21	14		
T4	19	18	1		
Regional lymph node metastasis				80.429	0.000
N0	46	1	45		
N1–3	46	44	2		
Distant metastasis				7.913	0.005
M0	85	38	47		
M1	7	7	0		
TNM stage				88.106	0.000
I	17	0	17		
II	29	0	29		
III	37	36	1		
IV	9	9	0		

### Consistency Analysis of CDH1 Methylation in PPW and Tumor Tissues From GC Patients

**Fig 1:**
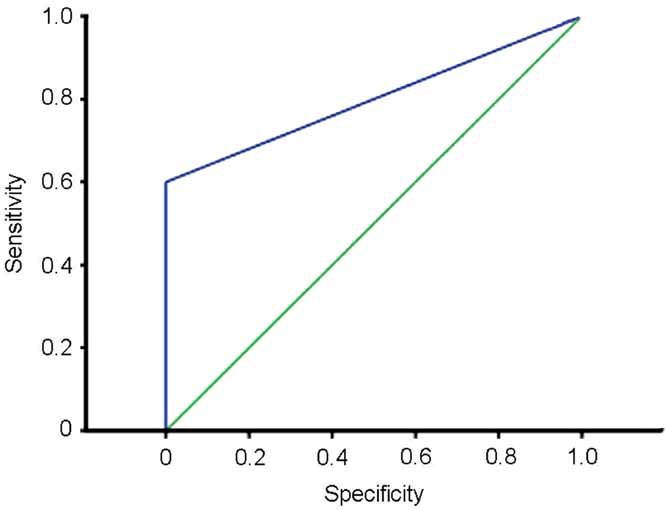
The diagnostic value of CDH1 methylation in PPW was determined by means of receiver-operating characteristic (ROC) curves, of which the Aζ value of ROC curve was 0.8 compared to GC tissues. Value of CDH1 methylation in PPW to diagnosis of gastric cancer is medium.

Of 92 GC patients, 45 specimens showed CDH1 methylation both in their PPW and tumor tissues. Using the results of CDH1 methylation in GC tissues as the golden standard, the diagnostic value of PPW was determined by means of receiver-operating characteristic (ROC) curves, of which the Aζ value of ROC curve was 0.8 compared to GC tissues (Fig. 1).

### Correlation Between the Results of CDH1 Methylation in PPW and Cytology

Of the 92 tumors, 39 (42.4%) showed a positive cytology. Peritoneal lavage cytology (PLC) was significantly related to the pathological findings. Overall 94.9% of patients with a positive PLC had a T3/T4 tumor and 100% of the patients with a positive PLC had a N-positive tumor (*P* < 0.001); in 76.9% of patients with a positive PLC, the tumor grade was low (*P* = 0.001). It was indicated that the rate of positive peritoneal wash samples increases proportionally when the tumor invades the deeper layers of the gastric wall or the lymph nodes, and when the tumor has lost differentiation. Of the 45 patients with a positive CDH1 methylation in PPW, 39 (86.7%) showed a positive cytology. And 100% of the patients with a positive PLC had a CDH1 methylation in PPW. The CDH1 methylation in PPW (*P* = 0.000, γ = 0.782) was closely correlated with the positive PLC.

### Effect of CDH1 Methylation in PPW on Prognosis of GC Patients

**Fig 2:**
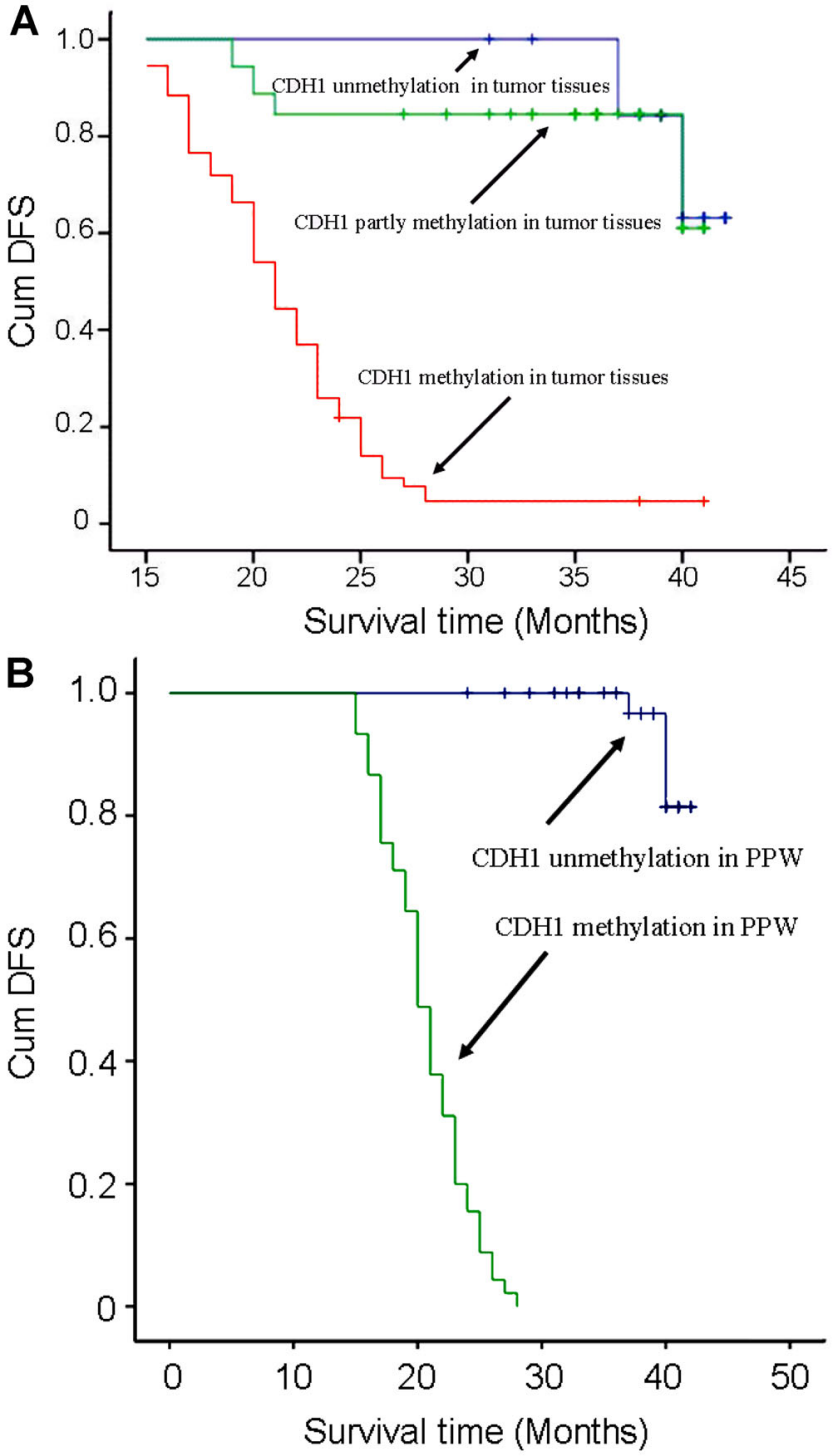
The relationship between the methylated status of CDH1 and survival. Kaplan–Meier analysis showed that there was a significant difference in disease-free survival (DFS) between the patients with or without methylated CDH1 in their tumor tissues (A) (χ^2^ = 55.467, *P* < 0.000), or in their PPW (B) (χ^2^ = 109.64, *P* < 0.000).

**Fig 3:**
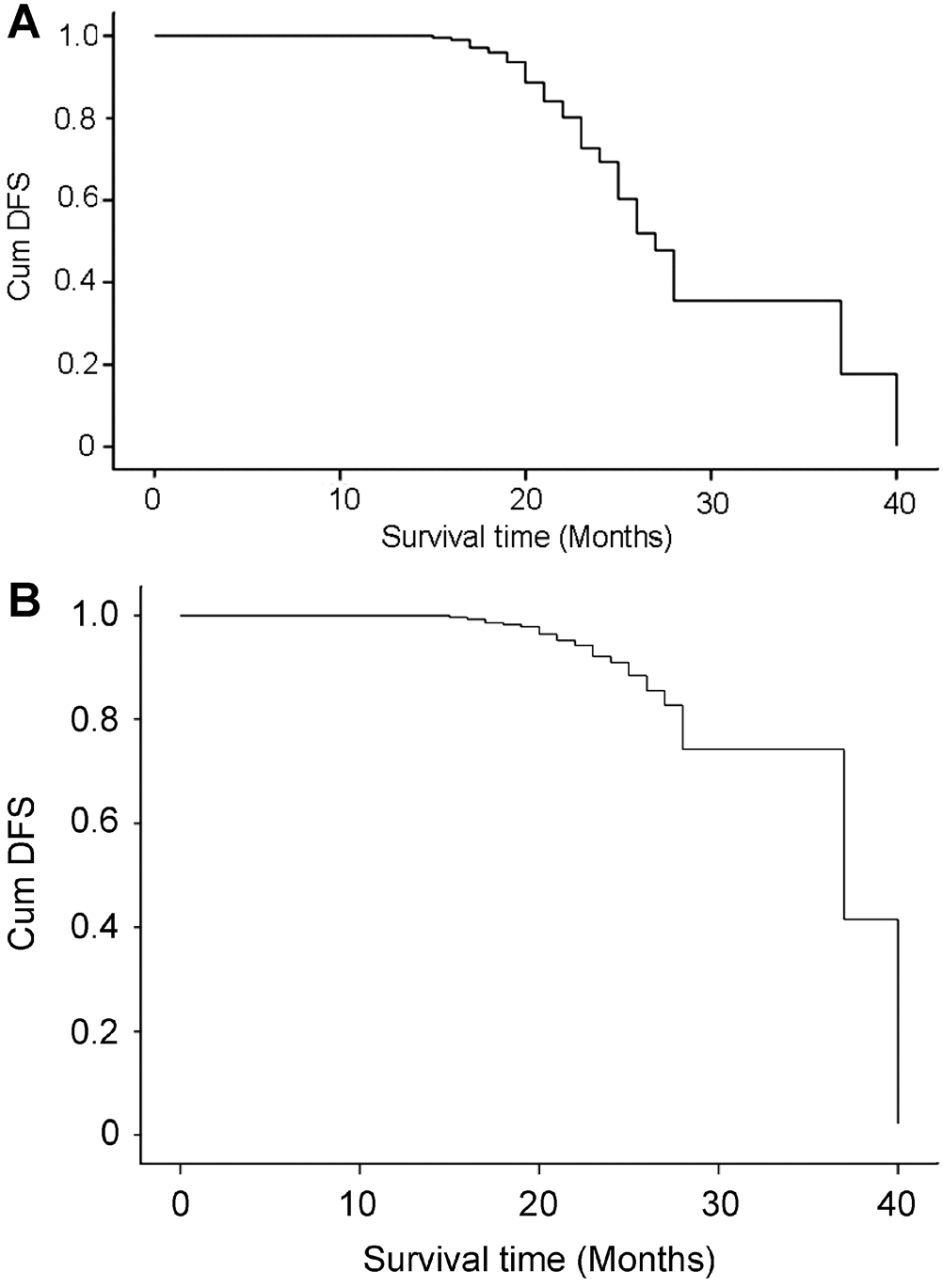
Cox regression model of survival analysis based on double factors (*CDH1* methylation and tumor clinical stage). Cox regression analysis showed CDH1 methylation in PPW was an independent risk factor for gastric cancer patients, with a remarkable decrease in DFS after postoperative 30 months. A: CDH1 methylation in tumor tissues; (B) CDH1 methylation in PPW.

All patients were followed-up after surgery treatment until the endpoint events occurred (tumor recurrence, metastasis, or death), then we analyzed the DFS. Up to June 30, 2011, compared to 4 progressed cases in 47 patients with CDH1 unmethylation, all of 45 patients with CDH1 methylation in PPW had progressed or died after 28 months' follow-up, with a marked decrease in DFS after postoperative 30 months. Median progression-free survival was only 20.93 months. Kaplan–Meier analysis showed that patients with CDH1 unmethylation in tumor tissues or in PPW exhibited an obvious survival advantage (both *P* = 0.000) (Fig. 2). It was demonstrated that the analysis of aberrant methylation in PPW is more significant than the analysis of aberrant methylation in primary tumor tissue. Meanwhile, Cox regression analysis revealed that patients with CDH1 unmethylation in their PPW had an independent survival advantage (*P* = 0.000; RR, 332.876; 95% CI, 21.705–5105.068) (Fig. 3). In addition, TNM stages could be considered as an influencing factor of prognosis in GC (RR, 307.058; 95% CI, 21.190–4449.397), only when the effect of CDH1 methylation was eliminated.

## DISCUSSION

Gastric cancer is still one of the most common malignancies worldwide, expected to be responsible for approximately 738,000 deaths in 2008 over the world 26. Previous evidences indicate that gastric cancer is the result of various genetic and epigenetic alterations of oncogenes, tumor suppressor genes, DNA repair genes, cell-cycle regulating proteins and cell adhesion molecules 27–30. DNA hypermethylation, as one of the most common epigenetic molecular alterations in human tumors, play an important role in the screening, early diagnosis and prognosis of cancer 31–33.

In GC, the inactivation of CDH1 by aberrant promoter methylation has been demonstrated 23. CDH1, a metastasis suppressor gene, locates on chromosome 16q22.1 and encodes calcium-dependent cellular adhesion molecule E-cadherin. Reduced expression of E-cadherin, or loss of E-cadherin function, is regarded as one of the main molecular events involved in the disorder of the intercellular adhesion system, triggering cancer invasion and metastasis 12–16, 34. Recent studies have found that the methylation of CDH1 promoter 5′-CpG island may be one of the leading causes to its inactivation 13–23. It is demonstrated that CDH1 promoter 5′-CpG island is methylated to varying degrees in choriocarcinoma 35, prostatic carcinoma 18, 36, malignant melanoma 19, non-small cell lung cancer 20, hepatocarcinoma 21, and gastric cancer 23, of which hypermethylation is implicated in the epigenetic silencing of the CDH1 gene, leading to reduced or absent E-cadherin expression 12–25, 34–36. Caldeira et al. 37 reported that high frequency of abnormal CDH1 methylation occurred in infiltrating breast cancers and it contributed to a decrease in E-cadherin expression. Ling et al. 22 have proved that down-regulated expression of CDH1 is closely correlated with histologic type, invasive depth, lymph node metastasis, and distant metastasis of esophageal cancer. Thus, it seems reasonable to presume that aberrant methylation of CDH1 promoter 5′-CpG island may be an early event involved in the pathogenesis of tumor, and is closely correlated with the prognosis of tumor 13–24, 34–37.

In present study, by utilizing real-time MSP to detect the CDH1 promoter methylation of GC tissues, we found that a much higher percentage of CDH1 methylation in GC tissues (81.5%, 75/92). On the contrast, CDH1 methylation promoter was not observed in the gastric mucosal tissues from 48 patients with chronic gastritis and 40 normal persons, suggesting that CDH1 methylation may contribute to the pathogenesis of GC. Furthermore, the rate of CDH1 methylation was 63.0% in GC tissues from the patient in stage I/II, which is much lower than that in stage I/II (100.0%) (*P* < 0.05), it is showed CDH1 methylation may be used in evaluating the malignant degree of the tumor. GC patients with N0 lymph node metastasis also presented a marked lower rate of CDH1 methylation, demonstrated its correlation to the development of GC. Meanwhile, our survival analysis validated CDH1 methylation may be a candidate as a prognostic marker for GC patients.

As we known, GC presents a high rate of abdomen metastasis. More than a century since Paget developed the theory of seed and soil, it was found that free tumor cells in peritoneal washes in some GC patients and considered these cells as the important factors of abdomen metastasis 38–40. However, few evidences reveal that how to free tumor cells penetrate the peritoneal mesothelial barrier and replant in abdomen, the mechanism is still unclear. It was supposed during the development of cancer, CDH1 hypermethylation of free tumor cells in abdomen brings about reduced expression of E-cadherin that inhibits intercellular adhesion and makes tumor cells easy to penetrate basement membrane into cancer-adjacent tissues and vessels, facilitates abdomino plantation of tumor cells. From that point, we sought to detect aberrant methylated DNA of cells in preoperative peritoneal washes from GC patients and to evaluate whether the detection of aberrant methylation is correlated with oncogenesis. Results indicated the percentage of CDH1 methylation in preoperative peritoneal washes was 48.9%, lower than that of other studies. We speculated it may because peritoneal washes contained some non-tumor cells, and we did not use laser capture microdissection to purify target cells, which remarkably affected the rate of CDH1 methylation.

We further analyzed the correlations between CDH1 methylation in PPW from GC patients and clinicopathologic parameters, then the results demonstrated that CDH1 methylation in preoperative peritoneal washes was significant correlated with tumor size, differentiation, clinical stage, invasive depth, lymphatic and venous invasion, lymph node metastasis, and distant metastasis (all *P* < 0.05). However, no correlation with gender, age, tumor site, and infection of *H. pylori* was found. These results were consistent with most literature, indicated that CDH1 methylation, not only in tumor tissues, but also in PPW, had significant correlation with tumor progression 41, 42. Kague et al. 43 detected 95% samples with CDH1 methylation had been infected by *H. pylori*, but we did not find any correlation between CDH1 methylation and *H. pylori* infection. It is necessary to investigate these controversial results and explain the exact mechanism.

In the present study CDH1 methylation in preoperative peritoneal washes was significant correlated with abdomen metastasis, and these GC patients with CDH1 hypermethylation demonstrated a poor prognosis. The following-up showed, compared to 4 progressed cases of 47 patients with CDH1 unmethylation, all of 45 patients with CDH1 methylation had progressed or died after 28 months' follow-up. Cox regression analysis revealed that these patients with CDH1 unmethylation in their peritoneal washes had an independent survival advantage (*P* = 0.000). CDH1 methylation exerted more remarkable effect on DFS for GC patients in contrast to clinical stage. Compelling study showed 5-year-survival rate of CDH1 methylated patients and CDH1 unmethylated patients were 35% and 67%, respectively. All these evidences suggest CDH1 hypermethylation predicts a poor prognosis.

It was observed CDH1 methylation in tumor tissues from GC patients whose PPW were detected CDH1 methylation. Using ROC curves, we determined that the diagnostic value of preoperative peritoneal washes was medium, in spite of little significance in early diagnosis of GC, may play an important role in predicting the progression and prognosis of GC.

In summary, aberrant methylation of CDH1 promoter 5′-CpG island was a frequent molecular event in GC. The detection of CDH1 methylation in PPW could be very applicable guidance for the diagnosis of tumor invasion and metastasis, also for prediction of progression and prognosis in GC.

## References

[1] Wang XQ, Yan H, Terry PD (2011). Interactions between CagA and smoking in gastric cancer. World J Gastroenterol.

[2] Alfaro EE, Lauwers GY: (2011). Early gastric neoplasia: Diagnosis and implications. Adv Anat Pathol.

[3] Qiao XT, Gumucio DL: (2011). Current molecular markers for gastric progenitor cells and gastric cancer stem cells. J Gastroenterol.

[4] Ibáñez de Cáceres I, Cairns P: (2007). Methylated DNA sequences for early cancer detection, molecular classification and chemotherapy response prediction. Clin Transl Oncol.

[5] Fiegl H, Elmasry K: (2007). Cancer diagnosis, risk assessment and prediction of therapeutic response by means of DNA methylation markers. Dis Markers.

[6] Ahmed IA, Pusch CM, Hamed T (2010). Epigenetic alterations by methylation of RASSF1A and DAPK1 promoter sequences in mammary carcinoma detected in extracellular tumor DNA. Cancer Genet Cytogenet.

[7] Leung WK, To KF, Man EP (2005). Quantitative detection of promoter hypermethylation in multiple genes in the serum of patients with colorectal cancer. Am J Gastroenterol.

[8] Rouprêt M, Hupertan V, Yates DR (2008). A comparison of the performance of microsatellite and methylation urine analysis for predicting the recurrence of urothelial cell carcinoma, and definition of a set of markers by Bayesian network analysis. BJU Int.

[9] Feng Q, Hawes SE, Stern JE (2007). Promoter hypermethylation of tumor suppressor genes in urine from patients with cervical neoplasia. Cancer Epidemiol Biomarkers Prev.

[10] Ibanez de Caceres I, Battagli C, Esteller M (2004). Tumor cell-specific BRCA1 and RASSF1A hypermethylation in serum, plasma, and peritoneal fluid from ovarian cancer patients. Cancer Res.

[11] Hwang SH, Kim KU, Kim JE (2011). Detection of HOXA9 gene methylation in tumor tissues and induced sputum samples from primary lung cancer patients. Clin Chem Lab Med.

[12] Li M, Zhang P: (2009). The function of APC/CCDH1 in cell cycle and beyond. Cell Div.

[13] Masterson J, O'Dea S: (2007). Posttranslational truncation of E-cadherin and significance for tumour progression. Cells Tissues Organs.

[14] Czyzewska J, Guzinska-Ustymowicz K, Ustymowicz M (2010). The expression of E-cadherin-catenin complex in patients with advanced gastric cancer: Role in formation of metastasis. Folia Histochem Cytobiol.

[15] Kim J, Hong SJ, Park JY (2010). Epithelial-mesenchymal transition gene signature to predict clinical outcome of hepatocellular carcinoma. Cancer Sci.

[16] Celebiler Cavusoglu A, Kilic Y (2009). Predicting invasive phenotype with CDH1, CDH13, CD44, and TIMP3 gene expression in primary breast cancer. Cancer Sci.

[17] Jeong DH, Youm MY, Kim YN (2006). Promoter methylation of p16, DAPK, CDH1, and TIMP-3 genes in cervical cancer: Correlation with clinicopathologic characteristics. Int J Gynecol Cancer.

[18] Florl AR, Steinhoff C, Muller M (2004). Coordinate hypermethylation at specific genes in prostate carcinoma precedes LINE-1 hypomethylation. Br J Cancer.

[19] Harbst K, Staaf J, Masback A (2010). Multiple metastases from cutaneous malignant melanoma patients may display heterogeneous genomic and epigenomic patterns. Melanoma Res.

[20] Kim DS, Kim MJ, Lee JY (2007). Aberrant methylation of E-cadherin and H-cadherin genes in nonsmall cell lung cancer and its relation to clinicopathologic features. Cancer.

[21] Matsumura T, Makino R, Mitamura K: (2001). Frequent downregulation of E-cadherin by genetic and epigenetic changes in the malignant progression of hepatocellular carcinomas. Clin Cancer Res.

[22] Ling ZQ, Li P, Ge MH (2011). Hypermethylation-modulated down-regulation of CDH1 expression contributes to the progression of esophageal cancer. Int J Mol Med.

[23] Barber M, Murrell A, Ito Y (2008). Mechanisms and sequelae of E-cadherin silencing in hereditary diffuse gastric cancer. J Pathol.

[24] Berx G, van Roy F: (2009). Involvement of members of the cadherin superfamily in cancer. Cold Spring Harb Perspect Biol.

[25] Ling ZQ, Tanaka A, Li P (2010). Microsatellite instability with promoter methylation and silencing of hMLH1 can regionally occur during progression of gastric carcinoma. Cancer Lett.

[26] Dikshit RP, Mathur G, Mhatre S (2011). Epidemiological review of gastric cancer in India. Indian J Med Paediatr Oncol.

[27] Piazuelo MB, Epplein M, Correa P: (2010). Gastric cancer: An infectious disease. Infect Dis Clin North Am.

[28] Wu WK, Cho CH, Lee CW (2010). Dysregulation of cellular signaling in gastric cancer. Cancer Lett.

[29] Oguma K, Oshima H, Oshima M: (2010). Inflammation, tumor necrosis factor and Wnt promotion in gastric cancer development. Future Oncol.

[30] Saikawa Y, Fukuda K, Takahashi T (2010). Gastric carcinogenesis and the cancer stem cell hypothesis. Gastric Cancer.

[31] Herceg Z, Vaissière T: (2011). Epigenetic mechanisms and cancer: An interface between the environment and the genome. Epigenetics.

[32] Tycko B: (2011). Cancer epigenetics and targeted therapies. Oncology (Williston Park).

[33] Brait M, Sidransky D: (2011). Cancer epigenetics: Above and beyond. Toxicol Mech Methods.

[34] Nawijn MC, Hackett TL, Postma DS (2011). E-cadherin: Gatekeeper of airway mucosa and allergic sensitization. Trends Immunol.

[35] Xue WC, Chan KY, Feng HC (2004). Promoter hypermethylation of multiple genes in hydatidiform mole and choriocarcinoma. J Mol Diagn.

[36] Saha B, Kaur P, Tsao-Wei D (2008). Unmethylated E-cadherin gene expression is significantly associated with metastatic human prostate cancer cells in bone. Prostate.

[37] Caldeira JR, Prando EC, Quevedo FC (2006). CDH1 promoter hypermethylation and E-cadherin protein expression in infiltrating breast cancer. BMC Cancer.

[38] Matsusaka S, Chìn K, Ogura M (2010). Circulating tumor cells as a surrogate marker for determining response to chemotherapy in patients with advanced gastric cancer. Cancer Sci.

[39] Lurje G, Schiesser M, Claudius A (2010). Circulating tumor cells in gastrointestinal malignancies: Current techniques and clinical implications. J Oncol.

[40] Takeuchi H, Kitagawa Y: (2010). Circulating tumor cells in gastrointestinal cancer. J Hepatobiliary Pancreat Sci.

[41] Hiraki M, Kitajima Y, Koga Y (2011). Aberrant gene methylation is a biomarker for the detection of cancer cells in peritoneal wash samples from advanced gastric cancer patients. Ann Surg Oncol.

[42] Hiraki M, Kitajima Y, Sato S (2010). Aberrant gene methylation in the peritoneal fluid is a risk factor predicting peritoneal recurrence in gastric cancer. World J Gastroenterol.

[43] Kague E, Thomazini CM, Pardini MI (2010). Methylation status of CDH1 gene in samples of gastric mucous from Brazilian patients with chronic gastritis infected by *Helicobacter pylori*. Arq Gastroenterol.

